# Interface of post-translational glycosylation and endoplasmic reticulum stress in melanoma: target to cancer cell sensitization to chemotherapeutic agents?

**DOI:** 10.1186/1753-6561-7-S2-P42

**Published:** 2013-04-04

**Authors:** Luiza HM Lourenço, Roger Chammas

**Affiliations:** 1 Department of Radiology, University of São Paulo, São Paulo, Brazil

## Background

Melanoma is the most lethal skin cancer, despite being less prevalent. Due to its lethality and resistance to a variety of known chemotherapeutic drugs, studies on melanoma are paramount. Tumor cells in general, and melanoma cells particularly, commonly present a disturbed metabolic rate, e.g, altered metabolism of reactive oxygen species and increased rates of protein synthesis. Altogether these perturbations would often trigger the Unfolded Protein Response (UPR); however, data from our laboratory showed that UPR markers are not constitutively expressed in tumorigenic cells. Our previous results suggest that melanoma cells have developed adaptative mechanisms to maintain an unstable equilibrium. Besides, glycosylation of tumor cells are commonly altered, due to differentiated expression rates of *N*-glycosylation enzymes, like *N*-acetylglucosaminyltransferase 5 (MGAT5). Our hypothesis is that the expression of MGAT5 (and/or MGAT5B) is part of an adaptive response to proteotoxic stresses, and on doing so they would serve as targets for sensitization of melanoma cells to UPR.

## Materials and methods

We treated non-tumorigenic melan-a cells and tumorigenic melanoma cells Tm1 and Tm5 *in vitro* with tunicamycin, a drug that prevents *N*-glycosylation, and swainsonine, a drug that prevents Golgi apparatus glycosylation.

## Results

Results showed that tumorigenic cells were more sensitive to both drugs and that they accumulated larger amounts of endoplasmic reticulum (ER) stress markers after treatments.

## Conclusions

These results show that Golgi apparatus glycosylation is important to maintain ER homeostasis, which is consistent with our hypothesis. Inhibition of MGAT5 expression would then be potentially useful for a combined treatment of melanoma cells with chemotherapeutic agents.

## Financial support

FAPESP and CAPES.

